# Case Report: Beyond metabolism: subclinical hypothyroidism associated with chronic alveolar hypoventilation

**DOI:** 10.3389/fmed.2026.1759084

**Published:** 2026-03-12

**Authors:** Sai Prasad, Shreya Sharma, Shaily Agrawal, Jay Nagda, Vidhi Parmar, Deep Bavaria, Darpan Kothia, Vidhi Dhaduk, Priyanshi Nada, Harsh Vardhan

**Affiliations:** 1 Department of Internal Medicine, S Nijalingappa Medical College Navanagar, Bagalkote, Karnataka, India; 2 Department of Internal Medicine, Hamdard Institute of Medical Sciences and Research, New Delhi, India; 3 Shri M P Shah Government Medical College, Jamnagar, Gujarat, India; 4 D Y Patil Medical College Navi Mumbai, Navi Mumbai, Maharashtra, India; 5 Department of Internal Medicine, Smt Shantabai Medical College and General Hospital, Amreli, India; 6 GMERS Sola, Ahmedabad, India; 7 Department of Internal Medicine, Hamdard Institute of Medical Sciences and Research, New Delhi, India

**Keywords:** hypoventilaion, OSA (obstructive sleep apnea), polysomnography, sub clinical hypothyroidism, thyroid dysfunction

## Abstract

**Background:**

Subclinical hypothyroidism, characterized by elevated thyroid-stimulating hormone levels with normal thyroid hormone levels, is frequently overlooked as a cause of respiratory dysfunction. While thyroid disorders are known to affect multiple organ systems, their role in precipitating chronic alveolar hypoventilation remains under-recognized in clinical practice.

**Case presentation:**

We report the case of a 48-year-old man with a body mass index (BMI) of 24.2 kg/m^2^ who presented with a three-month history of excessive daytime sleepiness, morning headache, and mild exertional breathlessness. Physical examination revealed no obvious signs suggestive of thyroid or primary respiratory pathologies. Arterial blood gas analysis demonstrated compensated type II respiratory failure with hypercapnia (pCO₂, 62 mmHg) and compensatory metabolic alkalosis (HCO₃^−^, 33 mEq/L). Pulmonary function tests revealed mildly decreased respiratory muscle strength, with a maximum inspiratory pressure (MIP) of 50 cmH₂O (45% predicted) and a maximum expiratory pressure (MEP) of 70 cmH₂O (47% predicted). Polysomnography confirmed sleep-related hypoventilation with an apnea-hypopnea index (AHI) of 3.2 events/h, oxygen desaturation index (ODI) of 2.8 events/h, and oxygen saturation nadir of 86%, effectively excluding obstructive sleep apnea (OSA). Thyroid function tests revealed subclinical hypothyroidism with elevated TSH (8.7 μIU/mL) but normal free thyroxine (T4) (1.2 ng/dL) and triiodothyronine (T3) (110 ng/dL) levels. Elevated anti-thyroid peroxidase antibody levels (120 IU/mL) suggested autoimmune thyroiditis.

**Management and outcome:**

Levothyroxine (50 μg/day) was initiated with a target TSH of 0.4–2.5 μIU/mL as per the European Thyroid Association guidelines, along with lifestyle modifications, including breathing exercises and moderate aerobic activity. After 2 months, the TSH level decreased to 2.1 μIU/mL. Complete normalization of gas exchange was achieved within 3 months (pCO₂: 44 mmHg, HCO₃^−^: 26 mEq/L, pH: 7.41, TSH: 2.6 μIU/mL), with resolution of all symptoms. The patient’s body weight remained stable throughout the follow-up period. Sustained improvement with no recurrence was documented at 6 months and 1 year.

**Conclusion:**

This case highlights the important temporal association between subclinical hypothyroidism and chronic alveolar hypoventilation. Although causality cannot be definitively established, the complete resolution following levothyroxine therapy suggests a clinically relevant relationship. This emphasizes the importance of thyroid function screening in patients with unexplained respiratory compromise after the exclusion of common causes.

## Introduction

Subclinical hypothyroidism is characterized by elevated thyroid-stimulating hormone (TSH) levels and normal free thyroxine (T4) and triiodothyronine (T3) concentrations. This condition affects approximately 4–10% of the general population, with a higher prevalence observed in women and older adults (1). Although often asymptomatic or presenting with mild symptoms, subclinical hypothyroidism can manifest with various systemic complications that extend beyond the commonly recognized metabolic disturbances.

The respiratory system is particularly susceptible to thyroid hormone deficiency, although this association remains underrecognized in clinical practice. Thyroid hormones play a crucial role in maintaining respiratory function via multiple mechanisms. They regulate the ventilatory response to hypoxia and hypercapnia, influence respiratory muscle strength and endurance, and affect the overall metabolic rate, which impacts oxygen consumption and carbon dioxide production (2, 3). Consequently, even mild thyroid dysfunction can impair respiratory mechanics and gas exchange.

Chronic alveolar hypoventilation represents a state of inadequate ventilation, leading to elevated arterial carbon dioxide levels (hypercapnia) and subsequent respiratory acidosis. While obesity hypoventilation syndrome, neuromuscular disorders, and obstructive sleep apnea are commonly attributed to obesity, endocrine etiologies, particularly thyroid dysfunction, are frequently overlooked (4). The respiratory manifestations of hypothyroidism include reduced ventilatory drive, respiratory muscle weakness, and altered lung mechanics, all of which may contribute to hypoventilation and respiratory complications.

Early identification of subclinical hypothyroidism as a potential contributor to chronic alveolar hypoventilation is clinically significant, as it represents a potentially reversible condition with a favorable prognosis when appropriately managed (1, 5). However, the subtle presentation of subclinical hypothyroidism often leads to a delayed diagnosis, with patients undergoing extensive evaluations for alternative respiratory and neurological causes before the endocrine etiology is considered.

This case report documents a temporal association between subclinical hypothyroidism and chronic alveolar hypoventilation, highlighting the importance of comprehensive evaluation, including thyroid function testing, in unexplained respiratory compromise.

## Case presentation

A 48-year-old man presented to the pulmonology outpatient department with a three-month history of excessive daytime sleepiness, morning headaches, and mild exertional breathlessness. He reported loud snoring during sleep, as noted by his spouse. The patient denied any history of chest pain, orthopnea, paroxysmal nocturnal dyspnea, or lower limb edema. There was no history of smoking, alcohol consumption, or occupational exposure to pulmonary irritants in the patient.

### Physical examination

At presentation, prior to the diagnosis of subclinical hypothyroidism, the patient appeared comfortable at rest. The vital signs were as follows: blood pressure, 128/78 mmHg; heart rate, 72 beats/min; respiratory rate, 16 breaths/min; and oxygen saturation, 94% on room air. His body mass index (BMI) was 24.2 kg/m^2^ (height 172 cm, weight 72 kg), and neck circumference was 38 cm. Cardiovascular examination revealed normal heart sounds without murmurs. Respiratory examination revealed bilateral equal air entry without adventitious sounds. Abdominal examination findings were unremarkable. Neurological examination showed no focal deficits, and there were no peripheral signs of hypothyroidism, such as dry skin, coarse hair, delayed tendon reflexes, or periorbital edema, at the time of initial presentation.

## Investigations

Arterial blood gas (ABG) analysis on room air demonstrated compensated type II respiratory failure, The elevated pCO₂ with compensatory increase in HCO₃^−^ and near-normal pH indicated chronic respiratory acidosis with metabolic compensation, consistent with chronic alveolar hypoventilation (see Table 1).

**Table 1:** Arterial blood gas (ABG) analysis.

Parameter	Value
pH	7.38 (7.35–7.45)
pCO₂	62 mmHg (35–45 mmHg)
pO₂	64 mmHg (80–100 mmHg)
HCO₃^−^	33 mEq/L (22–26 mEq/L)
SpO₂	94% (95–100%)

Thyroid function tests revealed elevated TSH levels with normal free T4 and T3 levels confirmed the diagnosis of subclinical hypothyroidism. Positive anti-thyroid peroxidase (anti-TPO) antibodies suggested Hashimoto’s thyroiditis as the underlying etiology of subclinical hypothyroidism. No goiter was identified on physical examination or imaging. Repeat TSH testing after 2 weeks confirmed persistent elevation (8.5 μIU/mL), excluding transient TSH elevation. Biotin supplementation, which can interfere with thyroid assays, was not used by the patient (see Table 2).

**Table 2:** Thyroid function tests.

Parameter	Value
TSH	8.7 μIU/mL (0.4–4.0 μIU/mL)
Free T4	1.2 ng/dL (0.8–1.8 ng/dL)
Free T3	110 ng/dL (80–180 ng/dL)
Anti-TPO antibodies	120 IU/mL (<35 IU/mL)

### Pulmonary function tests

Spirometry revealed normal lung volumes and flow rates, with an FEV₁/FVC ratio of 0.82, excluding obstructive or restrictive lung disease. Respiratory muscle strength assessment revealed:

**Table:** 

Parameter	Value
MIP (Maximum Inspiratory Pressure)	50 cmH₂O (45% predicted, normal >80 cmH₂O)
MEP (Maximum Expiratory Pressure)	70 cmH₂O (47% predicted, normal >100 cmH₂O)

Measurements were performed with the patient in sitting position using a flanged mouthpiece.

### Polysomnography

Overnight polysomnography was performed to assess sleep-disordered breathing. Continuous transcutaneous CO₂ monitoring was used throughout the study according to the American Academy of Sleep Medicine (AASM) criteria for sleep hypoventilation assessment. Key findings included:

**Table:** 

Parameter	Value
Total Sleep Time	412 min
Sleep Efficiency	86%
AHI (Apnea-Hypopnea Index)	3.2 events/h (normal <5)
ODI (Oxygen Desaturation Index)	2.8 events/h
Oxygen Saturation Nadir	86%
Mean SpO₂	93%
TcCO₂ > 55 mmHg	>10% of total sleep time
Sleep Stages	N1: 8%, N2: 52%, N3: 18%, REM: 22%

A normal AHI effectively excluded obstructive sleep apnea as the primary cause of respiratory symptoms. The presence of sustained transcutaneous CO₂ > 55 mmHg for >10% of total sleep time met the AASM criteria for sleep-related hypoventilation. Sleep architecture demonstrated a relatively preserved distribution of sleep stages.

### Additional laboratory investigations

The complete blood count, comprehensive metabolic panel, serum electrolytes (including phosphate, potassium, and magnesium), creatine kinase, and vitamin D levels were within normal limits. Serum sodium: 140 mEq/L, potassium: 4.2 mEq/L, phosphate: 3.5 mg/dL, magnesium: 2.1 mg/dL. These results exclude electrolyte imbalances as contributors to respiratory muscle weakness.

### Imaging studies

Chest radiography revealed normal lung fields with a normal cardiac silhouette and no evidence of pulmonary congestion or infiltrates. High-resolution computed tomography of the chest revealed no interstitial lung disease, parenchymal abnormalities, or structural lesions. Magnetic resonance imaging of the brainstem showed no abnormalities, excluding central causes of hypoventilation, such as brainstem lesions or Chiari malformation.

### Differential diagnosis

Several potential causes of chronic alveolar hypoventilation were systematically evaluated and excluded.

1. Obesity Hypoventilation Syndrome (OHS): OHS was considered based on the patient’s history of snoring and daytime hypersomnolence. However, the patient’s BMI of 24.2 kg/m^2^ was well below the diagnostic threshold for OHS (BMI ≥ 30 kg/m^2^). Additionally, a neck circumference of 38 cm is within the normal limits. The low AHI (3.2 events/h) and absence of significant nocturnal oxygen desaturation patterns, which are typical of OHS, further excluded this diagnosis.
2. Neuromuscular Disorders: Conditions such as myasthenia gravis, muscular dystrophies, or motor neuron disease were considered. However, the patient had no clinical features of neuromuscular disease (no muscle wasting, fasciculations, or progressive weakness in other muscle groups). Neurological examination was normal, with preserved reflexes and no bulbar symptoms. The improvement in respiratory muscle strength following levothyroxine therapy argues against a primary neuromuscular etiology.
3. Diaphragm Dysfunction: Although formal diaphragmatic ultrasound, fluoroscopy, or sniff testing was not performed, isolated diaphragmatic dysfunction was considered less likely. The absence of paradoxical abdominal breathing, lack of hemidiaphragm elevation on imaging, and the presence of both inspiratory and expiratory muscle weakness suggested more generalized ventilatory pump involvement rather than isolated diaphragmatic pathology. However, objective diaphragmatic testing was not performed, and therefore diaphragmatic dysfunction cannot be definitively excluded.
4. Electrolyte Imbalances: Hypophosphatemia, hypokalemia, and hypomagnesemia can contribute to respiratory muscle weakness. However, a comprehensive metabolic panel demonstrated normal serum levels of phosphate (3.5 mg/dL), potassium (4.2 mEq/L), and magnesium (2.1 mg/dL), effectively excluding these as contributing factors.
5. Central Nervous System Pathology: Brainstem lesions, Arnold-Chiari malformation, and other structural CNS abnormalities were excluded by normal magnetic resonance imaging (MRI) of the brainstem.
6. Chronic Obstructive Pulmonary Disease: The patient had no history of smoking or occupational exposure to respiratory irritants. Spirometry showed a normal FEV₁/FVC ratio (0.82), and high-resolution CT of the chest revealed no emphysematous changes or chronic bronchitis, excluding COPD.

## Treatment

Based on the diagnostic findings and systematic exclusion of alternative etiologies, a clinical diagnosis of subclinical hypothyroidism with associated chronic alveolar hypoventilation was made.

### Pharmacological management

Levothyroxine therapy was initiated at a dose of 50 μg/day. The treatment target was set at TSH 0.4–2.5 μIU/mL, in accordance with the European Thyroid Association (ETA) guidelines for the management of subclinical hypothyroidism. These guidelines recommend targeting a TSH level within the lower half of the normal reference range (0.4–2.5 μIU/mL) for symptomatic patients with subclinical hypothyroidism and positive anti-TPO antibodies, as was the case in our patient. This target differs from the less stringent goal of TSH < 3 μIU/mL sometimes used in asymptomatic patients, as our patient had significant end-organ effects warranting more aggressive treatment.

### Non-pharmacological interventions

Concurrent with levothyroxine therapy, the patient was advised lifestyle modifications, including (1) daily breathing exercises (incentive spirometry and diaphragmatic breathing techniques) to improve respiratory muscle conditioning and (2) moderate aerobic exercise (30 min of brisk walking, 5 days per week) to enhance overall cardiopulmonary fitness. These interventions serve as adjunctive measures to support respiratory function during thyroid hormone replacement.

### Ventilatory support

Given the chronic compensated nature of the respiratory failure (as evidenced by metabolic compensation on ABG) and the absence of acute decompensation, noninvasive ventilatory support was not initiated. The patient was clinically stable with adequate oxygenation on room air (SpO₂ 94%) and was closely monitored as an outpatient during the initiation of levothyroxine therapy.

## Outcome and follow-up

### Two-month assessment

In accordance with the ETA guidelines recommending TSH reassessment 2 months after treatment initiation (rather than the previously mentioned 3-month timeline), thyroid function was evaluated at this interval. The TSH level decreased to 2.1 μIU/mL, approaching the target range. The patient reported subjective improvement in daytime alertness and a reduction in morning headaches.

### Three-month assessment

Complete arterial blood gas normalization was achieved at 3 months of therapy:

**Table:** 

Parameter	Value
pH	7.41
pCO₂	44 mmHg
HCO₃^−^	26 mEq/L

Repeat thyroid function testing showed a TSH level of 2.6 μIU/mL, within the target range. All respiratory symptoms resolved completely. The patient’s body weight remained stable at 72 kg (BMI 24.2 kg/m^2^), indicating that weight change did not confound respiratory improvement.

### Long-term follow-up

At six-month follow-up, the patient remained asymptomatic. Repeat ABG and PFT results were within normal limits. Respiratory muscle strength improved to MIP 95 cmH₂O and MEP 135 cmH₂O, representing 85 and 90% of the predicted values, respectively. Levothyroxine was administered at a dose of 50 μg/day.

One-year follow-up demonstrated sustained clinical and biochemical improvements, with no recurrence of respiratory symptoms. The patient continued on levothyroxine 50 μg/day, with TSH maintained at 2.3 μIU/mL.

## Discussion

This case demonstrates a temporal association between subclinical hypothyroidism and chronic alveolar hypoventilation, with complete resolution following levothyroxine therapy. While the observed improvement suggests a clinically meaningful relationship, it is important to emphasize that temporal association does not establish causality, and residual confounding cannot be fully excluded.

Although respiratory complications of overt hypothyroidism are well established, the respiratory effects of subclinical hypothyroidism remain less clearly defined. Prior studies have suggested that even mild thyroid dysfunction may influence ventilatory physiology. Ansarin et al. demonstrated altered ventilatory physiology in both subclinical and overt hypothyroidism, with differences in end-tidal CO₂ compared with healthy controls, suggesting altered ventilatory regulation even in milder thyroid dysfunction states (5). Similarly, spirometric studies have demonstrated subtle pulmonary function alterations in subclinical hypothyroidism, although the clinical significance of these findings remains uncertain (6).

The physiological basis of respiratory dysfunction in hypothyroidism is multifactorial. Thyroid hormones play a key role in modulating ventilatory drive through central chemoreceptor sensitivity to hypoxia and hypercapnia. Classic studies in overt hypothyroidism have demonstrated impaired ventilatory responses to hypercapnia and hypoxia, supporting the biological plausibility of thyroid hormone influence on respiratory control (3). In addition, hypothyroidism is associated with respiratory muscle weakness. Studies evaluating respiratory muscle performance in hypothyroid patients have demonstrated reduced inspiratory and expiratory muscle strength, which improves following thyroid hormone replacement (4).

Additional mechanisms may include altered skeletal muscle metabolism, mitochondrial dysfunction, and impaired neuromuscular transmission. Although these mechanisms are well described in overt hypothyroidism, it is plausible that milder forms of these abnormalities may occur in subclinical hypothyroidism. Furthermore, alterations in lung mechanics, including reduced lung compliance and surfactant abnormalities, have been described in hypothyroid states and may contribute to hypoventilation physiology (7).

The presence of elevated anti-TPO antibodies in this patient confirmed autoimmune thyroiditis as the underlying etiology. Currently, there is limited evidence directly linking thyroid autoimmunity itself to ventilatory dysfunction independent of thyroid hormone levels. Most literature supports antibody positivity primarily as a marker of autoimmune thyroid disease and predictor of progression to overt hypothyroidism rather than an independent contributor to respiratory dysfunction (1, 8). This represents an area for future investigation.

The patient’s clinical presentation was notable for the absence of classic hypothyroid features, highlighting that subclinical hypothyroidism may occasionally present with isolated organ dysfunction. The presenting symptoms of excessive daytime sleepiness, morning headaches, and exertional dyspnea are consistent with chronic nocturnal hypoventilation and CO₂ retention. Importantly, more common causes of chronic hypercapnia were systematically evaluated and excluded. Obesity hypoventilation syndrome was unlikely given the normal BMI and low AHI. Neuromuscular disease was not supported clinically or radiologically. Electrolyte disturbances were excluded biochemically. Structural central nervous system causes were excluded by imaging. The absence of smoking history, normal spirometry, and normal chest imaging excluded chronic obstructive pulmonary disease.

The complete normalization of arterial blood gases and symptoms following levothyroxine therapy supports a clinically meaningful association. However, concurrent lifestyle interventions, including breathing exercises and aerobic conditioning, may have contributed to improvement. The stable body weight throughout follow-up reduces weight change as a confounding factor. Nonetheless, concurrent non-pharmacological interventions—specifically daily breathing exercises and a structured aerobic exercise programme—must be acknowledged as significant potential confounders, as these independently improve respiratory muscle strength and cardiopulmonary fitness. The relative contribution of levothyroxine therapy versus lifestyle modifications cannot be definitively determined from this single case. The rapid and sustained improvement following thyroid hormone replacement suggests that thyroid dysfunction likely played a significant role in this patient’s clinical presentation, but controlled study designs would be required to isolate this effect.

Respiratory failure due to overt hypothyroidism is well documented, including reports of reversible diaphragmatic dysfunction and hypoventilation syndromes responding to thyroid replacement therapy (7, 9). However, reports describing chronic hypercapnic respiratory failure associated with subclinical hypothyroidism remain limited. Current clinical guidelines emphasize treatment of symptomatic subclinical hypothyroidism, particularly in patients with TSH > 8–10 μIU/mL or positive thyroid antibodies, supporting the therapeutic approach used in this case (8, 10).

This case contributes to the limited body of evidence suggesting that subclinical hypothyroidism may have clinically significant respiratory manifestations in select patients. The complete reversibility following levothyroxine therapy highlights the importance of considering thyroid dysfunction in the evaluation of unexplained chronic hypoventilation after exclusion of more common etiologies.

Existing literature has well established respiratory dysfunction in overt hypothyroidism, including ventilatory drive impairment, respiratory muscle weakness, and reversible diaphragmatic dysfunction (3, 4, 7, 9). In contrast, respiratory manifestations of subclinical hypothyroidism remain incompletely characterized, with most studies demonstrating only subtle physiological abnormalities without clear clinical respiratory failure (5, 6). Reports describing chronic hypercapnic respiratory failure or objectively documented nocturnal hypoventilation in subclinical hypothyroidism are extremely limited.

This case adds to the literature by demonstrating objectively documented sleep-related hypoventilation using transcutaneous CO₂ criteria, clinically significant ventilatory pump weakness, and complete normalization of gas exchange and respiratory muscle strength following levothyroxine therapy. While causality cannot be established, this case highlights the need for further investigation into respiratory manifestations of subclinical hypothyroidism.

This case has several strengths. The diagnosis was supported by objectively documented sleep-related hypoventilation meeting AASM transcutaneous CO₂ criteria, systematic exclusion of alternative etiologies using biochemical, spirometric, radiological, and polysomnographic assessments, and a sustained one-year follow-up demonstrating durable clinical and biochemical improvement. The stable body weight throughout the follow-up period reduces the likelihood that weight change confounded the observed respiratory improvement.

Several limitations must be acknowledged. Baseline diaphragmatic function testing using ultrasound or fluoroscopy was not performed, and supine versus upright forced vital capacity measurements were not obtained; therefore, diaphragmatic or isolated neuromuscular contributors cannot be fully excluded. Follow-up polysomnography after treatment would have provided objective confirmation of hypoventilation resolution but was not available. Importantly, concurrent non-pharmacological interventions—specifically daily breathing exercises (incentive spirometry and diaphragmatic breathing) and a structured aerobic exercise programme—must be acknowledged as significant potential confounders. These interventions independently improve respiratory muscle strength and cardiopulmonary fitness, and it is not possible to attribute the observed improvement exclusively to levothyroxine therapy. Finally, as a single case report, generalizability remains limited, and further prospective studies are needed to clarify the independent relationship between subclinical hypothyroidism and ventilatory dysfunction.

## Conclusion

This case demonstrates a temporal association between subclinical hypothyroidism and chronic alveolar hypoventilation, with resolution of hypercapnia and symptoms following levothyroxine therapy in this patient. While causality cannot be established from a single case, these findings suggest thyroid function testing may be reasonable in selected patients with unexplained chronic hypoventilation after exclusion of common causes. Further studies are needed to clarify this relationship.

## Data Availability

The original contributions presented in the study are included in the article/supplementary material, further inquiries can be directed to the corresponding author.
